# Comparison of gastrointestinal side effects from different doses of azithromycin for the treatment of gonorrhoea

**DOI:** 10.1093/jac/dkac118

**Published:** 2022-04-13

**Authors:** Jason J Ong, Ivette Aguirre, Magnus Unemo, Fabian Y S Kong, Christopher K Fairley, Jane S Hocking, Eric P F Chow, Warittha Tieosapjaroen, Jenny Ly, Marcus Y Chen

**Affiliations:** Central Clinical School, Monash University, Melbourne, Australia; Melbourne Sexual Health Centre, Alfred Hospital, Melbourne, Australia; Faculty of Infectious and Tropical Diseases, London School of Hygiene and Tropical Medicine, London, UK; Melbourne Sexual Health Centre, Alfred Hospital, Melbourne, Australia; WHO Collaborating Centre for Gonorrhoea and other STIs, Örebro University, Örebro, Sweden; Institute for Global Health, University College London (UCL), London, UK; Melbourne School of Population and Global Health, The University of Melbourne, Melbourne, Australia; Central Clinical School, Monash University, Melbourne, Australia; Melbourne Sexual Health Centre, Alfred Hospital, Melbourne, Australia; Melbourne School of Population and Global Health, The University of Melbourne, Melbourne, Australia; Central Clinical School, Monash University, Melbourne, Australia; Melbourne Sexual Health Centre, Alfred Hospital, Melbourne, Australia; Melbourne School of Population and Global Health, The University of Melbourne, Melbourne, Australia; Melbourne Sexual Health Centre, Alfred Hospital, Melbourne, Australia; Melbourne Sexual Health Centre, Alfred Hospital, Melbourne, Australia; Central Clinical School, Monash University, Melbourne, Australia; Melbourne Sexual Health Centre, Alfred Hospital, Melbourne, Australia

## Abstract

**Objectives:**

Azithromycin is commonly used to treat *Neisseria gonorrhoeae*. We compared its gastrointestinal side effects using 1 g single, 2 g single or 2 g split (i.e. 1 g plus 1 g 6–12 h later) dosing, representing our clinic’s changing guidelines over the study period.

**Methods:**

We recruited consecutive sexual health clinic patients who received azithromycin (and 500 mg ceftriaxone) for uncomplicated gonorrhoea. Each patient received a text message 48 h after their attendance to complete a questionnaire.

**Results:**

Patients received 1 g single (*n *= 271), 2 g single (218) or 2 g split (105) doses. Vomiting was less common for 1 g versus 2 g single dose [1.1% versus 3.7%; risk difference (RD): −2.6%; 95% CI: −0.2 to −5.4] and 2 g split versus 2 g single dose (0.9% versus 3.7%; RD: −2.8%; 95% CI: −0.3 to −5.8). Nausea was less common for 1 g versus 2 g single dose (13.7% versus 43.1%; RD: −29.5%; 95% CI: −21.7 to −37.2) and 2 g split versus 2 g single dose (16.4% versus 43.1%; RD: −26.8; 95% CI: −17.2 to −36.3). Diarrhoea was less common for 1 g versus 2 g single dose (25.5% versus 50.9%; RD: −25.5%; 95% CI: −17.0 to −33.9) and 2 g split versus 2 g single dose (30.9% versus 50.9%; RD: −20.0; 95% CI: −9.1 to −30.9). Almost all were willing to retake the same dosing for gonorrhoea in the future: 97% for 1 g single; 94% for 2 g single; and 97% for 2 g split dose.

**Conclusions:**

Azithromycin 2 g split dose for gonorrhoea resulted in significantly less vomiting, nausea and diarrhoea than a 2 g single dose.

## Introduction

Over time, gonorrhoea has become resistant to multiple classes of antibiotics. Ceftriaxone is currently the only antibiotic that remains fully effective against gonorrhoea. When occasional cases of treatment failure with ceftriaxone emerged over the last one to two decades, dual antimicrobial therapy was introduced using ceftriaxone (250 mg to 1 g single intramuscular dose) with azithromycin (1–2 g single oral dose) internationally.^1–3^ These regimens additionally eradicate concomitant *Chlamydia trachomatis* infections and some concurrent *Mycoplasma genitalium* infections. However, the increasing resistance to azithromycin now challenges its inclusion in dual therapy for gonorrhoea.^4^ Some countries have recently returned to ceftriaxone monotherapy as recommended first-line treatment for uncomplicated gonorrhoea, e.g. the UK and the USA (ceftriaxone 1 g monotherapy and ceftriaxone 500 mg monotherapy, respectively), when a chlamydial infection has been excluded.^5,6^ The 2020 European gonorrhoea guideline recommends ceftriaxone 1 g plus azithromycin 2 g as a single dose. If side effects are anticipated, azithromycin can be given as a 2 g split dose, that is 1 g then 1 g after 6–12 h.^7^ However, ceftriaxone 1 g monotherapy is recommended as an option for anorectal gonorrhoea in well-controlled settings and circumstances (including lack of ceftriaxone resistance, use of test of cure, and administration of doxycycline if *C. trachomatis* infection has not been excluded).^7^

The main rationale for maintaining azithromycin in the dual therapy is to avoid treatment failure with ceftriaxone monotherapy, which has been sporadically documented, particularly with pharyngeal infection,^8^ and predicted to be more common based on pharmacokinetic/pharmacodynamic (PK/PD) modelling.^9^ In Australia, ceftriaxone 500 mg intramuscularly is given with azithromycin 1 g orally to treat genital and anorectal gonorrhoea and with azithromycin 2 g single dose to treat pharyngeal gonorrhoea.^10^ The azithromycin 2 g dose appears more effective than the azithromycin 1 g dose for treating ceftriaxone-resistant gonorrhoea or when azithromycin is used alone in the absence of azithromycin resistance.^11^ However, gonococcal isolates are now increasingly considered to be resistant to azithromycin internationally.^4^

At Melbourne Sexual Health Centre (MSHC), the largest public sexual health centre in Australia, we administer azithromycin 2 g single dose, as recommended for pharyngeal gonorrhoea, but are concerned about a possible increase in gastrointestinal side effects.^12,13^ There are no studies comparing the gastrointestinal side effects using different dosing of azithromycin, particularly for the 2 g split dose. Bergan *et al*.^14^ found that after 12 h, blood levels post a 1 g dose were about 10% of peak levels, and Hoffler *et al*.^15^ found that after 6 and 12 h, blood levels after a 1 g dose were 10%–25% of peak levels. Thus, taking the second 1 g dose 6–12 h post the first 1 g dose may reduce side effects since high blood levels are minimized. Notably, studies confirming a higher cure rate for gonococcal infections with azithromycin 2 g split dose treatment compared with azithromycin 1 g single dose therapy are lacking.

We aimed to compare the side-effect profile of azithromycin 1 g single dose, 2 g single dose and 2 g split dose to treat uncomplicated urogenital and extragenital gonorrhoea. Azithromycin was used together with ceftriaxone 500 mg, as per Australian STI Management Guidelines.^10^ We were interested in gastrointestinal side effects (i.e. vomiting after giving each azithromycin dose, nausea, diarrhoea) using the different azithromycin dosing.

## Materials and methods

### Study population

This was a quasi-experimental study (before-and-after design) where consecutive patients attending MSHC received treatment for uncomplicated urogenital and extragenital gonorrhoea in three phases: (1) azithromycin 1 g single dose for both urogenital and extragenital gonorrhoea (3 June 2019 to 24 May 2020); (2) azithromycin 2 g single dose for pharyngeal gonorrhoea (27 August 2019 to 24 May 2020; individuals with urogenital or anal gonorrhoea continued to receive 1 g single dose in this period); and (3) azithromycin 2 g split dose for pharyngeal gonorrhoea (25 May 2020 to 21 May 2021). Pharmacy staff at MSHC invited patients diagnosed with gonorrhoea to participate in the survey when they dispensed the antibiotics. The survey was an anonymous online survey hosted on Research Electronic Data Capture (REDCap), a secure web-based software platform for research studies.^16^ Verbal consent was received, and each patient was given an information leaflet and received a unique, secure link to the online questionnaire via text message 48 h after receiving the ceftriaxone injection at MSHC.

Inclusion criteria included: (1) aged 18 years or over; (2) diagnosed with *N. gonorrhoeae*; (3) had reasonable command of English and could provide informed consent; and (4) treated with ceftriaxone *plus* azithromycin for gonorrhoea. Pharmacy staff provided all patients (regardless of whether they agreed to participate or not) with standard advice on how to take the azithromycin tablets when giving out the medication; this advice included information about taking the medicine straight after food and commonly occurring side effects. Patients are advised to be sexually abstinent for 7 days for all dosing regimens.

### Sample size

We anticipated that approximately 1% of patients taking a 1 g dose of azithromycin would experience vomiting.^17^ Studies assessing the tolerability of a 2 g oral dose of azithromycin indicate that up to 7% will experience vomiting when given on an empty stomach,^12,13^ but this could be reduced after food. This study compared the proportion of patients experiencing gastrointestinal side effects between the three azithromycin doses. A sample size of approximately 210 in each group provided 80% power to detect a difference in vomiting between the 1 g dose and the 2 g dose if the proportion with vomiting was 1% and 6% or more, respectively (alpha 5%).We also examined a third group whereby patients given 2 g azithromycin split their dose, i.e. 1 g followed 6–12 h later by 1 g. We initially planned to recruit 210 individuals into this third group, but due to the impact of the COVID-19 pandemic causing reductions in gonorrhoea cases and delays in recruitment, we decided to stop recruitment at 100 patients for the 2 g split dose during the study when the data showed a significant difference between the three groups.

### Ethics

Ethics approval was received from the Alfred Hospital Ethics Committee (256/19).

### Survey measures

Participants were sent a text message 48 h after being prescribed the medication and asked whether they had experienced any nausea, diarrhoea or vomiting since taking the antibiotic. Participants given the 2 g split dose were not sent a prompt to take the second dose. We asked about the following: the time between eating food and taking the antibiotics; the time between taking the first 1 g dose and the next 1 g dose (for the 2 g split dose group); the time between taking the antibiotics; the experience of any side effects; and the duration of the side effects. We used a Likert scale (where 1 = very, very mild and 5 = most severe) to measure the severity of each side effect. Besides gastrointestinal side effects, we asked whether participants experienced any other side effects. Finally, we asked whether they would accept the same treatment if they needed treatment for gonorrhoea again. Concurrent medications were extracted from their current pharmacy records, and the site of gonorrhoea infection was extracted from their electronic health record. Test of cure was not conducted because failure with ceftriaxone is rare.

### Statistical analysis

We used descriptive statistics to summarize the proportion of people experiencing various side effects: vomiting, nausea and diarrhoea. We calculated the 95% CIs for the difference in proportions using exact binomial (Clopper–Pearson) CIs. We also present an adjusted risk difference (RD) including gender, age and weight as potential confounders. A *P* value of <0.05 was considered to be statistically significant. Box plots were created to summarize the time to side effects. All analysis was performed using Stata (Stata Statistical Software, Release 17, College Station, TX, USA; StataCorp LLC).

## Results

### Study population

A total of 1067 individuals agreed to receive the follow-up SMS, with 594 consenting and completing the survey: 271 (azithromycin 1 g single dose), 218 (2 g single dose) and 105 (2 g split dose). All individuals in the 2 g split dose group reported that they took the second dose. The median time interval between doses was 8 h (IQR: 6.5–11.5). Most (94%; 558/594) were male with a mean age of 32.0 years (SD 9.1). Further details of the study population are shown in Table 1.

**Table 1.** Characteristics of the patients with gonorrhoea administered differing doses of azithromycin (*N = *594)

Characteristic	1 g single dose (*N *= 271)	2 g single dose (*N *= 218)	2 g split dose (*N *= 105)
Site of gonorrhoea infection, *n* (%)^a^			
Pharyngeal	132 (48.7)	169 (77.5)	96 (91.4)
Genital	67 (24.7)	20 (9.2)	2 (1.9)
Anorectal	134 (49.4)	73 (33.5)	21 (20.0)
Age, years	** **	** **	** **
Mean (SD)	31.6 (9.0)	32.3 (9.2)	33.2 (8.9)
Median (IQR)	30 (26–35)	30 (26–36)	31 (27–36)
Gender, *n* (%)	** **	** **	** **
Male	247 (91.1)	208 (95.4)	93 (88.6)
Female	13 (4.8)	8 (3.7)	3 (2.9)
Other	6 (2.2)	1 (0.5)	9 (8.6)
Mean BMI (SD)	24.4 (4.1)	24.6 (4.3)	25.3 (5.6)
Concurrent antibiotics given on the day, *n* (%)^b^	14 (5.2)	6 (2.8)	3 (2.7)
Sexual identity, *n* (%)			
MSM	221 (81.5)	190 (87.2)	93 (88.6)
Heterosexual	30 (11.1)	19 (8.7)	3 (2.9)
Bisexual	10 (3.7)	7 (3.2)	9 (8.6)
Other infections diagnosed, *n* (%)^c^	92 (33.9)	69 (31.7)	37 (35.2)

*N *= number of the participants included in the analyses.

a The total is >100% because individuals may have had a multisite infection.

b Antibiotics administered in addition to azithromycin. 1 g single dose: two received amoxicillin, one received cefalexin, five received benzathine penicillin, five received doxycycline, one received metronidazole and one received flucloxacillin; 2 g single dose: one received amoxicillin, three received doxycycline, one received phenoxymethylpenicillin and one received flucloxacillin; 2 g split dose: one received penicillin, one received roxithromycin and one received cefalexin.

c Other infections diagnosed included chlamydia, syphilis, HIV and *M. genitalium*.

### Gastrointestinal side effects

Table 2 shows the side-effect profiles for the three dosing groups. Vomiting was less common with azithromycin 1 g versus 2 g single dose (1.1% versus 3.7%; RD: −2.6%; 95% CI: −0.2 to −5.4). Nausea was less common with azithromycin 1 g versus 2 g single dose (13.7% versus 43.1%; RD: −29.5%; 95% CI: −21.7 to −37.2) and diarrhoea was less common with azithromycin 1 g versus 2 g single dose (25.5% versus 50.9%; RD: −25.5%; 95% CI: −17.0 to −33.9).

**Table 2.** Side-effect profile of different azithromycin dosing

Side effect	1 g single dose (*N *= 271) % (95% CI)	2 g single dose (*N *= 218) % (95% CI)	2 g split dose (*N *= 105) % (95% CI)	2 g single versus 1 g single dose		2 g single versus 2 g split dose	
				Absolute RD (95% CI)	Adjusted RD^a^ (95% CI)	Absolute RD (95% CI)	Adjusted RD^a^ (95% CI)
Vomiting	1.1 (0.2–3.2)	3.7 (1.6–7.1)	0.9 (0.0–5.0)	2.6 (0.2–5.4)	2.9 (0.1–5.6)	2.8 (0.3–5.8)	4.6 (1.0–10.1)
Nausea	13.7 (9.8–18.3)	43.1 (36.4–50.0)	16.4 (10.0–24.6)	29.5 (21.7–37.2)	28.5 (20.8–36.2)	26.8 (17.2–36.3)	32.4 (15.8–49.0)
Diarrhoea	25.5 (20.4–31.1)	50.9 (44.1–57.7)	30.9 (22.4–40.4)	25.5 (17.0–33.9)	25.9 (17.4–34.4)	20.0 (9.1–30.9)	10.3 (12.8–33.5)

*N *= number of participants.

a Adjusted for gender, age and weight.

Vomiting was less common with azithromycin 2 g split versus 2 g single dose (0.9% versus 3.7%; RD: −2.8%; 95% CI: −0.3 to −5.8). Nausea was less common with azithromycin 2 g split versus 2 g single dose (16.4% versus 43.1%; RD: −26.8%; 95% CI: −17.2 to −36.3) and diarrhoea was less common with azithromycin 2 g split versus 2 g single dose (30.9% versus 50.9%; RD: −20.0%; 95% CI: −9.1 to −30.9). Our results did not change when adjusted for gender, age and weight.

When comparing 2 g split with 1 g single dose, there was no significant difference in reports of vomiting (0.9% versus 1.1%; RD: −0.2%; 95% CI: −2.0 to 1.6) or nausea (16.4% versus 13.7%; RD: 0.8%; 95% CI: −5.5 to 7.0). However, there was significantly more diarrhoea for 2 g split compared with 1 g single dose (30.9% versus 25.5%; RD: 9.4; 95% CI: 1.2–17.6). When adjusted for gender, age and weight, the difference in diarrhoea was no longer significant (95% CI: −6.4 to 40.6).

### Timing of gastrointestinal side effects

Only 11 people reported vomiting, so we did not perform any statistical analysis of the differences in the timing between taking azithromycin and onset of vomiting. In terms of the severity of vomiting (1 = very, very mild and 5 = worse vomiting imaginable), of those who took the 1 g dose (*n *= 3), two had a score of 1 and one had a score of 4. For those who took the 2 g single dose (*n *= 8), three had a score of 2, three had a score of 3 and two had a score of 4. No score was reported for the person who took the 2 g split dose.

**Figure 1.**
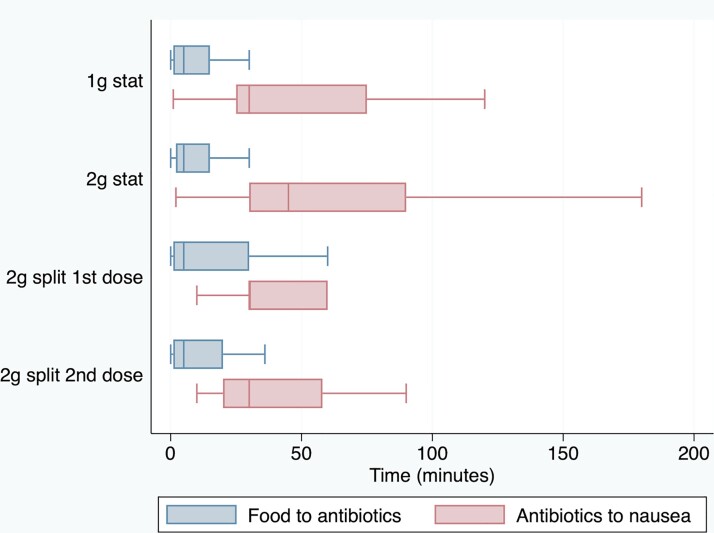
Time between taking food and azithromycin, and time from taking azithromycin to nausea. The lines within the boxes refer to the medians. The upper and lower bounds of the boxes refer to the 75th and 25th percentiles, respectively. The whiskers refer to the values up to 1.5 times the IQR. This figure appears in colour in the online version of *JAC* and in black and white in the print version of *JAC*.

Figure 1 summarizes the timing between taking food and azithromycin (blue box plots) among those with nausea. For the1 g single dose, the median time was 5 min (IQR 1–15). For the 2 g single dose, the median time was 5 min (IQR 2–15). For the 2 g split dose, the median time for the first dose was 5 min (IQR 2–14) and the median time for the second dose was 5 min (IQR 1.5–20). Figure 1 also summarizes the timing between taking azithromycin and nausea (red box plots). For the 1 g single dose, the median time was 30 min (IQR 25–75). For the 2 g single dose, the median time was 45 min (IQR 30–90). For the 2 g split dose, the median time for the first dose was 30 min (IQR 30–60) and the median time for the second dose was 30 min (IQR 20–58).

**Figure 2.**
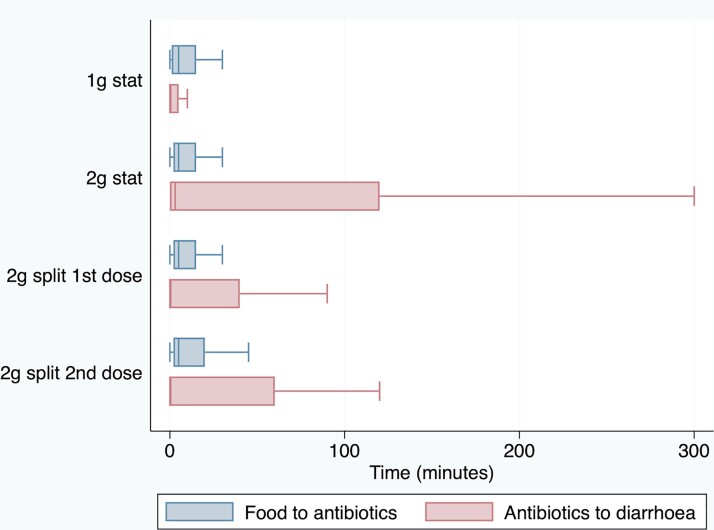
Time between taking food and azithromycin, and time from taking azithromycin to diarrhoea. The lines within the boxes refer to the medians. The upper and lower bounds of the boxes refer to the 75th and 25th percentiles, respectively. The whiskers refer to the values up to 1.5 times the IQR. This figure appears in colour in the online version of *JAC* and in black and white in the print version of *JAC*.

Figure 2 summarizes the timing between taking food and azithromycin (blue box plots) among those with diarrhoea. For the 1 g single dose, the median time was 5 min (IQR 1–15). For the 2 g single dose, the median time was 5 min (IQR 2–15). For the 2 g split dose, the median time for the first dose was 7.5 min (IQR 1–20) and the median time for the second dose was 10 min (IQR 1–30). Figure 2 also summarizes the timing between taking azithromycin and diarrhoea (red box plots). For the 1 g single dose, the median time was 120 min (IQR 60–180). For the 2 g single dose, the median time was 120 min (IQR 60–120). For the 2 g split dose, the median time for the first dose was 75 min (IQR 60–120) and the median time for the second dose was 105 min (IQR 60–180).

For the 1 g single dose, the median number of diarrhoea episodes was 3 (IQR 2–4). For the 2 g single dose, the median number was 3 (IQR 2–4). For the 2 g split dose, the median number after the first dose was 2 (IQR 1–3) and the median number after the second dose was 2 (IQR 1–3). There was no difference between people who had food within 1 h and those who did not with regard to vomiting (*P *= 0.567), nausea (*P *= 0.959) or diarrhoea (*P *= 0.428).

Despite reported gastrointestinal side effects, almost all participants were willing to retake the same treatment dosing for gonorrhoea in the future: 97% (263/271; 95% CI: 94–99) for the 1 g single dose; 94% (206/218; 95% CI: 91–97) for the 2 g single dose; and 97% (99/102; 95% CI: 92–99) for the 2 g split dose.

## Discussion

In this study of 594 people treated for gonorrhoea, we found significant differences in the frequency of gastrointestinal side effects reported, depending on the dosing of azithromycin administered. Within this population, who also received a ceftriaxone 500 mg intramuscular single dose, increasing azithromycin from a 1 g single to a 2 g single dose resulted in more vomiting, nausea and diarrhoea. Splitting the 2 g dose (two 1 g doses 6–12 h apart) compared with the 2 g single dose significantly reduced all gastrointestinal side effects to levels similar to those seen with a 1 g single dose. Despite the high proportion of gastrointestinal side effects in this study population, almost all patients would still take the same medication if they were infected with gonorrhoea in the future.

Although there is a general move to remove azithromycin as first-line therapy from sexually transmitted infection (STI) guidelines in the USA,^5^ the UK^6^ and Europe,^7^ 2 g azithromycin is still recommended by Australian guidelines for treating oropharyngeal gonorrhoea^10^ and US guidelines recommend gentamicin plus 2 g azithromycin as an alternative when there is cephalosporin allergy.^5^ The European guideline also recommends 1 g ceftriaxone plus 2 g azithromycin for uncomplicated gonorrhoea in some situations, including the presence of ceftriaxone resistance, lack of test of cure or no administration of doxycycline if *C. trachomatis* infection has not been excluded.^7^ Ceftriaxone monotherapy has variable bioavailability in the oropharynx, which potentially could lead to more treatment failures and an increased likelihood of antimicrobial resistance.^18^

The frequency of vomiting, particularly in the first hour, is important for a drug with low oral bioavailability, such as azithromycin.^19^ Our finding of the low reports of vomiting (1% for 1 g single, 4% for 2 g single and 1% for 2 g split dose) is similar to the product monograph for azithromycin reporting that in adult patients (*n *= 904) 1.7% reported vomiting for 1 g single, 6.7% for 2 g single dose and 1% when patients received azithromycin (total 30 mg/kg) over 3 or 5 days.^17^ These findings are consistent with the theory that vomiting may be related to blood levels of azithromycin, so reducing peak levels would theoretically reduce this. In a study of 431 participants receiving a 2 g single dose of azithromycin for gonorrhoea, 7% reported vomiting.^13^ In another study of 202 participants receiving gentamicin with a 2 g single dose of azithromycin, 7.4% reported vomiting, and in 199 participants who received gemifloxacin with a 2 g single dose of azithromycin, 5% reported vomiting (or 10% when the safety population analysis was performed to include all who received the therapy).^12^ It is important to note that from these dual therapy studies (like our study), it is not possible to ascertain the contribution of azithromycin to gastrointestinal side effects separately from concomitant antibiotics. We did not find any significant difference between the timing of food and the report of vomiting within an hour of taking azithromycin, although our sample size may be too small to detect a statistically significant difference.

Our study also reported a high frequency of other gastrointestinal side effects. Azithromycin absorption may be suboptimal when patients have vomiting or diarrhoea, but we did not have data to examine the impact on the microbiological cure. Our reports of diarrhoea (26% for 1 g single, 51% for 2 g single and 31% for 2 g split dose) are higher compared with those reported in the product monograph (6.1% for 1 g single, 13.8% for 2 g single dose and 4%–5% for multiple-dose regimens over 3 to 5 days).^17^ This might be because there is no standard method for measuring the severity of diarrhoea. Furthermore, the addition of ceftriaxone may add to the reports of diarrhoea.^20^ Azithromycin has a prokinetic effect on the gastrointestinal system, explaining the high frequency of diarrhoea. Our study’s reports of nausea (14% for 1 g single, 43% for 2 g single, 16% for 2 g split dose) are also higher than the data from the product monograph (4.9% for nausea for 1 g single, 18.2% for 2 g single and 3%–4% for multiple-dose regimens over 3 to 5 days).^17^ This could also be due to the additional impact of taking ceftriaxone.^20^

The strength of our study is that we prospectively followed a large cohort of individuals treated for gonorrhoea who actively reported their experience of side effects. We minimized recall bias by sending the survey via text message 48 h after the patient took azithromycin. Our findings should be read in the light of several limitations. First, our study population is from a single clinic in Australia. Our results should be verified in studies of other populations. Second, there is a possibility of detection bias; those who experienced a side effect may have been more likely to respond to the survey. All participants were aware they were given azithromycin with no placebo as a control. Third, the exceptionally high acceptability to retake the same medication despite significant side effects may be due to social desirability bias or the perception that they had no other option to cure gonorrhoea. Finally, as this was not an experimental study design, we did not randomize patients so there could be unmeasured confounders between the three groups; nor did we mandate patients to have a test of cure, so we could not comment on whether different azithromycin regimens impacted microbiological cure.

In conclusion, we found differing side-effect profiles, particularly regarding nausea and diarrhoea, depending on the azithromycin dosing regimens in the treatment of gonorrhoea. Azithromycin 1 g single or 2 g split dose for the treatment of gonorrhoea resulted in significantly less vomiting, nausea and diarrhoea than 2 g single dose. Despite a high proportion of individuals who reported gastrointestinal side effects, almost all patients would still take the same medication if they were infected with gonorrhoea in the future.
